# ECMO during the COVID-19 pandemic: when is it unjustified?

**DOI:** 10.1186/s13054-020-03230-9

**Published:** 2020-08-17

**Authors:** Darryl Abrams, Roberto Lorusso, Jean-Louis Vincent, Daniel Brodie

**Affiliations:** 1 Columbia University College of Physicians & Surgeons/New York-Presbyterian Hospital, 622 W168th St, PH 8E, Rm 101, New York, NY 10032 USA; 2 Center for Acute Respiratory Failure, Columbia University Medical Center, New York, NY USA; 3 Cardiothoracic Surgery Department, Heart & Vascular Centre, Maastricht University Medical Centre, Cardiovascular Research Institute Maastricht, Maastricht, The Netherlands; 4 Department of Intensive Care, Erasme University Hospital, Université Libre de Bruxelles, Brussels, Belgium

The coronavirus disease 2019 (COVID-19) pandemic has led to a critical shortage of resources in the hardest-hit areas around the world [1]. Intensive care units (ICUs) overwhelmed by critically ill patients may create non-conventional ICU spaces and even consider triaging invasive mechanical ventilation to those most likely to benefit [2]. In the most severe cases of refractory hypoxemia, extracorporeal membrane oxygenation (ECMO) may be considered, as recommended by the World Health Organization for severe COVID-19. Early data suggest there may be a benefit from ECMO in certain patients with COVID-19-associated respiratory failure, though outcomes are likely to be highly dependent on patient selection and timing of ECMO initiation [3]. Whether certain phenotypes of COVID-19 (if present) have differential responses to and prognoses with ECMO remains to be determined [4]. An important question then is whether a resource-intensive therapy is warranted when systems are already strained [5].

The high severity of the respiratory failure in some patients with COVID-19 anticipates the need for ECMO in a large number of patients. However, circumstances that limit otherwise readily available resources raise the threshold for initiating more complex therapies. Therefore, in the context of the COVID-19 pandemic, adherence to evidence-based algorithms is necessary to optimize the allocation of limited resources. Every effort should be made to apply established, less invasive strategies, including prone positioning and optimization of volume status, prior to consideration of ECMO in these patients [6], but ECMO may still be required. In fact, the limited availability of ECMO, due in part to shortages in ECMO equipment and insufficient capacity at ECMO-capable centers, may lead to the unanticipated benefit of more widespread adoption of these proven therapies that often go underutilized [7].

Perhaps the initial question should not be when, but whether to use ECMO at all in the COVID-19 pandemic. Analyses have demonstrated a benefit from ECMO in severe forms of the acute respiratory distress syndrome (ARDS) [8], though such benefit comes at real costs, and not simply financial ones. In the case of a pandemic requiring crisis standards of care, every resource has the potential to become critical to the functioning of an ICU or the care of critically ill patients. Most prominently, staffing may emerge as a critical bottleneck. The use of ECMO taxes many resources, but none more so than staffing—increased nursing ratios, need for ECMO specialists, disproportionate medical provider time, not to mention other staff, such as respiratory or physical therapists, who would be needed elsewhere for the care of other patients [9]. Given that staffing may already be maximally strained, the excess resources needed for the ECMO patient will negatively and disproportionately impact the care of non-ECMO patients relative to the addition of another critically ill patient not receiving ECMO. During a crisis, ECMO may not be a zero-sum game. The inability to manage this strain will likely be greatest among lower-volume, less-experienced ECMO centers, providing rationale for the regionalization of ECMO [9], an approach which itself may be further limited by excess patient volume at all centers, resulting in suboptimal provision of care to ECMO patients in general.

In this context, can ECMO be justified in the epicenter of a pandemic? During non-pandemic times, in hospitals or regions with sufficient staffing reserves and provider availability, it may be understandable why clinicians might attempt ECMO in a candidate with a low, but acceptable, probability of benefit (e.g., a post-partum patient with refractory shock in multisystem organ failure). Yet, one would be hard-pressed to justify the same approach if it meant a tangible sacrifice in the care of other patients in whom there is greater likelihood of favorable outcomes. Effectively, at times of substantially increased strain on hospital and healthcare systems, there needs to be more judicious patient selection, reserving ECMO only for those patients who are most likely to derive benefit, assuming an acceptable level of care can be maintained for other patients, in an attempt to achieve the greatest benefit for the greatest number of patients—a utilitarian standard that may apply under crisis standards of care. Beyond withholding ECMO, the most dire of situations may seem to necessitate the withdrawal of ECMO from those in whom there is no perceived chance of meaningful recovery—regardless of the opinion of the patient or surrogate decision-maker [10, 11]. Triage committees may be helpful to help determine the allocation of resources under such circumstances [12].

The use of ECMO in a pandemic can be seen following a U-shaped curve (Fig. 1), rising as the number of cases rises, decreasing as resources become increasingly scarce, and possibly rising again as strain eases on the back-end of the crisis or trailing off as the number of patients qualifying for ECMO likewise tapers down. Of course, under the most extreme of circumstances (at the bottom of the curve), ECMO may have to be abandoned altogether [13]. Therein lies the key principle: the use of ECMO should not be considered in a vacuum; the consequences of choosing to initiate ECMO in a crisis are not just borne by that patient alone.

**Fig. 1:**
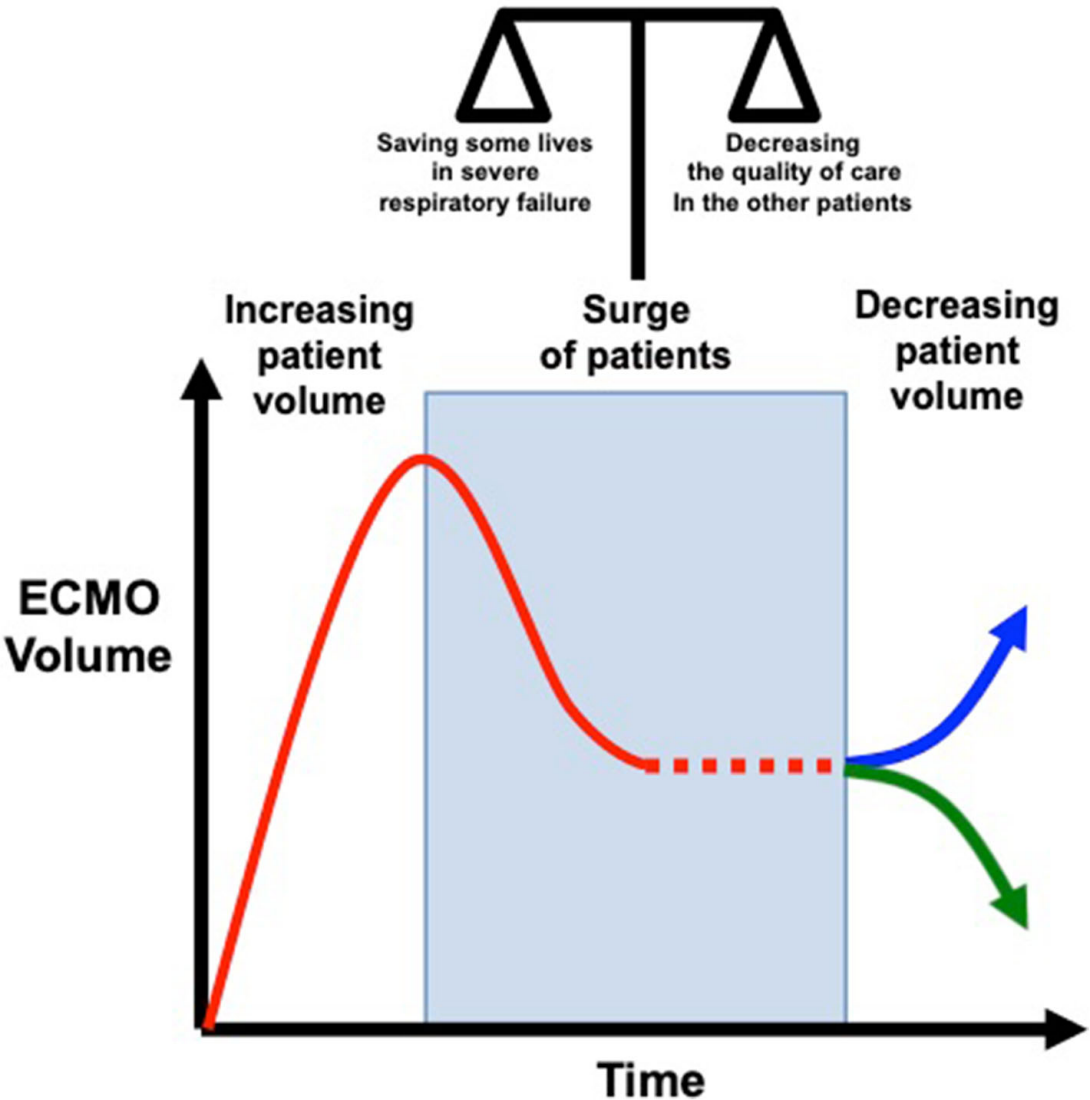
Potential curve of ECMO case volume during the COVID-19 pandemic. During surge conditions, ECMO usage will be variable (red dashed line), including the potential of being abandoned altogether. As the pandemic resolves and patient volume decreases, there may be increasing resource availability and ECMO use (blue arrow) or decreasing demand (green arrow)

## Data Availability

Not applicable
